# Research on the Reliability of a Core Control Unit of Highway Electromechanical Equipment Based on Virtual Sensor Data

**DOI:** 10.3390/s22207755

**Published:** 2022-10-13

**Authors:** Shan Lin, Mi Luo, Jun Niu, Hongke Xu

**Affiliations:** School of Electronic and Control Engineering, Chang’an University, Xi’an 710064, China

**Keywords:** traffic engineering, fatigue life, pavement vibration, printed circuit board, reliability

## Abstract

The printed circuit board (PCB) is the core control unit of electromechanical equipment. In order to determine the influence of the coupling vibration caused by vehicle–road interaction on the PCB reliability of roadside electromechanical equipment, first, the dynamic load of the vehicle tire is solved by establishing the dynamic model of a vehicle road. Then, the acceleration response data generated by road vibration are obtained by solving the road finite element model. Finally, the power density spectrum of the acceleration response is taken as input excitation, and the deformation response of the PCB under vehicle–road coupling vibration is analyzed. The experimental results show that when the vehicle is driving close to the roadside, the vibration caused by vehicle–road coupling will lead to a large deformation of the PCB, and the deformation value reaches 0.170 mm, which can cause structural damage to the PCB. This shows that the vehicle–road coupling vibration can affect the reliability of the roadside electromechanical equipment; thus, the optimal design of the PCB layout is created. After optimization, the first-order modal frequency of the PCB is increase by 5.4%, which reduces the risk of the components breaking away from the PCB substrate.

## 1. Introduction

In modern traffic, the operation and service of the highway cannot be separated from the support of all kinds of equipment in the electromechanical system [1]. However, the electromechanical equipment will inevitably go through the process of performance degradation and component damage in the service life. Qi’s research [2] shows that at a vibration level of 0.1 g^2^/Hz, it takes only 16 h and 19 min to cause an intermittent failure of the PCB. Moreover, this fault can become a permanent fault under the continuous action of random vibration. In the Fu’s study [3], the dynamic load power transferred by the vehicle to the road surface can reach a maximum of 0.208 g^2^/Hz. Thus, it can be seen that the random vibration caused by vehicle–road coupling can lead to the failure of the PCB. In particular, due to its long-term work in the complex traffic environment, the PCB in the electromechanical equipment is easily affected by vehicle–road coupling vibration. This effect can result in the accumulation of fatigue damage, causing structural deformation of the PCB [4]. As a result, the components break off, resulting in functional failure of the PCB, and affecting the normal operation of the equipment. Although some researchers have developed a system which can realize pavement vibration monitoring [5], there is some uncertainty about the influence of vehicle–road coupling vibration on the reliability of electromechanical equipment. In order to avoid economic losses, it is necessary to use finite element analysis to prove the concept [6,7]. To study the influence of vehicle–road coupling vibration on highway roadside electromechanical equipment PCB, it is necessary to study the two aspects of vehicle–road coupling vibration, along with the influence of vibration on PCB reliability, at the same time.

In most of the research analyzing the interaction between the vehicle and the road, the vehicle model is generally simplified, and the road is abstracted as a beam model to establish the vehicle–road dynamics equation [8,9,10,11,12]. The research results of Zhang et al. show that the influence of vehicle–road coupling vibration on the propagation of pavement cracks is very obvious [13]. In some cases, buildings near the road are often damaged due to continuous traffic vibration. This shows that the random vibration caused by vehicle–road coupling can spread along the road and affect the surrounding infrastructure [14]. This point is also mentioned in the research of Arabi [15] and Wang [16] through the establishment of a three-dimensional human–bus–road coupling vibration system; the analysis shows that the road vibration can affect human comfort. Currently, the main research direction of vehicle–road coupling vibration focuses on the damage of vehicle–road action on asphalt pavement and adjacent buildings, along with the influence of such vibrations on pedestrian comfort. However, the research is rarely related to the reliability of electromechanical equipment working in complex traffic environments under the long-term influence of random vibration.

By establishing the finite element model of electronic packaging devices, Qin [17] carried out a series of vibration fatigue experiments on the solder joints of electronic packaging devices, and modified the Steinberg model according to the research results. Rajaguru modeled and analyzed the vibration of the power electronic module (PEM) structure, taking the random vibration of the vehicle as the excitation, and drew the conclusion that the vibration has an obvious influence on the fatigue life of the electronic structure [18]. Similarly, with the road load as the incentive, the research results of Wang show that even the piezoelectric energy collector specifically used to collect road vibration energy will experience the problem of performance degradation under the continuous action of traffic load [19]. Muhammad [20] studied the remaining useful life (RUL) of electronic modules under harsh random vibrations and operating conditions, analyzed the effects of vibration on the storage conditions and soft faults of the PCB solder joints, and estimated the random vibration fatigue damage of solder joints with the help of finite element software. Prashanth [21] studied the vibration of the PCB using the finite element method, and determined the dynamic characteristics. At the same time, he used the finite element model to study the influence of boundary conditions on vibration response. When analyzing the reliability of electronic equipment in a vibration environment, most of the scholars focus analyzing the structural dynamic response of its internal PCB. This is because the PCB carries a large number of electronic components, and the connection between these components and the PCB substrate is often weak. Once these components fall off, it may lead to the loss of the whole PCB function, affecting the normal operation of the complete equipment.

With the continuous improvement in traffic capacity, the road traffic load is also increasing, and vibration, one of the most negative effects of traffic behavior, has caused great damage to the environment [22]. In recent years, with an increasing amount of electromechanical equipment installed on the roadside, some of this equipment has been used in a traffic environment to assist in its operation and management [23]. Therefore, in order to further improve the service life of roadside equipment and avoid serious failures, it is necessary to comprehensively consider the impact of traffic system coupling vibration on the operation of the equipment. However, considering that the vehicle–road coupling vibration is generally weak, it is uncertain whether this vibration will cause fatigue damage to the PCB in electromechanical equipment. To save costs, it is necessary to verify the feasibility of the method through simulation experiments in advance [24]. Based on the above analysis, this paper attempts to establish the road finite element model and the simplified the PCB model and then, through the virtual sensor in the finite element platform, to collect the road acceleration response data. Based on this, this paper studies the influence of random vibration caused by the interaction between the vehicle and the road on the operational reliability of roadside electromechanical equipment to provide a theoretical basis for subsequent real experiments.

## 2. Random Roughness Fitting of Road Surface

The excitation of the pavement system is the direct cause of the vehicle–road coupling vibration [25,26]. During the driving of the vehicle on the road, the existence of road roughness will generate random vibrations, which leads to continuous change in the vehicle tire force [27]. Therefore, road roughness is the basis for studying the road dynamic response [28]. Generally speaking, the road roughness is determined by its statistical characteristics, and the statistical characteristics of the road roughness can be described by the road power spectral density [29]. According to the “Fitting of Road Spectral Characteristics and Power Spectral Density” in GB/T 7031-2005, the given power spectral density fitting formula as expressed in Equation (1):

(1) $$G_{d}(n)=G_{d}(n_{0}){\left(\frac{n}{n_{0}}\right)}^{-w}$$

where n is the spatial frequency (m^−1^); $n_{0}$ is the reference spatial frequency, (0.1 m^−1^);$G_{d}(n_{0})$ is the road roughness coefficient (m^3^); and w is the index of fitting power spectral density.

In this paper, the trigonometric series method is used to simulate the random roughness of the road surface. The focus of the analysis is approximated by the random process of the target by a discrete spectrum, and then the road power spectrum is decomposed into sine waves with different amplitudes and frequencies using the discrete Fourier transform. The square of the amplitude of these sine waves after being divided by the bandwidth is the spectral density [30]. In other words, the road profile of any road can be represented by a set of discrete sine waves. Therefore, under the condition that the road frequency domain model is known, the amplitude of each sine wave can be calculated by its corresponding frequency spectral density, and its phase difference can be randomly generated by software.

Assuming that the vibration natural frequency range of the vehicle is $\left(f_{1},f_{u}\right)$, the upper and lower limits ($n_{1}$,$n_{u}$) of the effective spatial frequency of the road roughness power spectral density can be expressed as in Equation (2):

(2) $$n_{1}=\frac{f_{1}}{v},n_{u}=\frac{f_{u}}{v}$$

In order to avoid frequency aliasing when calculating the power spectral density, the sampling interval Δl should be satisfied with Equation (3):

(3) $$\Delta l\leq \frac{1}{2n_{u}}$$

If the power spectral density $G_{q}(n)$ of the road surface is known in the frequency range $\left[f_{1},f_{u}\right]$, the variance of the road roughness $\sigma_{z}^{2}$ can be expressed as in Equation (4)

(4) $$\sigma_{z}^{2}=\int_{f_{1}}^{f_{u}}G_{q}(n)dn$$

Divide $\left[f_{1},f_{u}\right]$ into m intervals, the power spectral density at the central frequency $f_{mid-i}(i=1,2,\ldots ,m)$ of each interval is taken as $G_{q}(f_{mid-i})$, and $G_{q}(f_{mid-i})\cdot \Delta m_{i}$ is used to replace the integral value of each interval; then Equation (4) can be approximately expressed as Equation (5):

(5) $$\sigma_{z}^{2}\approx \sum_{i=1}^{m}G_{q}(f_{mid-i})\cdot \Delta m_{i}$$

where $\Delta m_{i}$ is the interval length.

It is easy to observe that the larger the value of m, the closer the result of Equation (5) is to that of Equation (4). For the interval of each frequency $f_{mid-i}(i=1,2,\ldots ,m)$, the sine wave function, whose standard deviation is $\sqrt{G_{q}(f_{mid-i})\cdot \Delta m_{i}}$, can be expressed as Equation (6).

(6) $$y_{i}=\sqrt{2G_{q}(f_{mid-i})\cdot \Delta m_{i}}\sin(2\pi f_{mid-i}x+\theta_{i})$$

where x is the horizontal displacement of the road surface; $\theta_{i}$ is a random number that satisfies uniform distribution on [0,2π).

If the sine wave functions of each interval are superimposed, the random roughness of the road surface in the time domain can be expressed as Equation (7).

(7) $$y(x)=\sum_{i=1}^{m}\sqrt{2G_{q}(f_{mid-i})\cdot \Delta m_{i}}\sin(2\pi f_{mid-i}x+\theta_{i})$$

**Figure 1:**
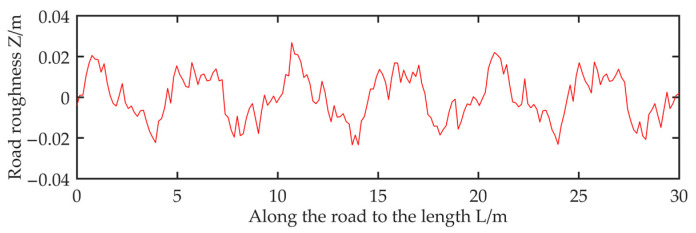
Variation of random unevenness of Class B pavement along the strike.

In this paper, according to the actual data of a road section, the length of the road section is 30 m and the vehicle speed is v = 20 m/s; the random roughness change of a Class B road surface is simulated by MATLAB, as shown in Figure 1.

## 3. Vehicle–Road Coupling Analysis

### 3.1. Analysis of Vehicle–Road Coupling Vertical Vibration

**Figure 2:**
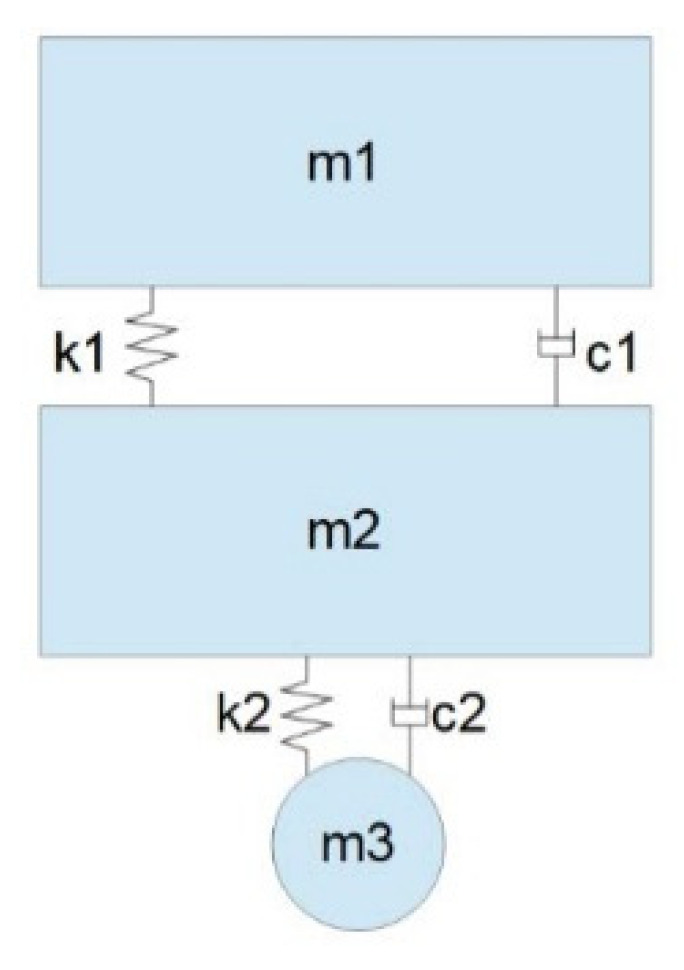
The 1/4 vehicle model.

Analyzing and establishing the dynamic model of vehicles running on the road is the basis of studying the coupling between the vehicle and the road system, but it is difficult to establish a model that can completely reflect all the characteristics of the vehicles. Therefore, the vehicle model is simplified, in most studies. According to the actual research needs, this paper selects a 1/4 vehicle model. The final 1/4 vehicle model is shown in Figure 2 [31,32,33].

In the Figure 2, m1 and m2 are the car body mass and wheel mass; the value of m3 is 0, which only indicates the position of the contact surface between the vehicle tire and the road surface; k1 represents suspension stiffness; c1 represents suspension damping; k2 represents wheel stiffness; and the value of c2 is 0, which ignores wheel damping.

The parameters of the common 10 t loader model are shown in Table 1.

Considering only the vertical displacement of the vehicle model when driving on the road, according to the D’Alembert’s principle:

(8) $$\sum_{i}\left(F_{i}-m_{i}a_{i}\right)=0$$

By stipulating that the vertical upward direction is positive, the vehicle body and wheels are analyzed. By assembling the model coefficient and response of the vehicle system [34], the vehicle vibration dynamic equilibrium equation can be established, as shown in the Equation (9).

(9) $$M_{v}\cdot {\ddot{X}}_{v}+C_{v}\cdot {\dot{X}}_{v}+K_{v}\cdot X_{v}=F_{v}$$

where $F_{v}$ is the force vector of the road to the vehicle; $M_{v}$, $C_{v}$, and $K_{v}$ represent the mass matrix, damping matrix, and stiffness matrix of the vehicle, respectively; ${\ddot{X}}_{v}$, ${\dot{X}}_{v}$, and $X_{v}$ represent the acceleration response, velocity response, and displacement response of the vehicle, respectively.

Similarly, the equilibrium equation of road vibration can be obtained as follows:

(10) $$M_{b}\cdot {\ddot{X}}_{b}+C_{b}\cdot {\dot{X}}_{b}+K_{b}\cdot X_{b}=F_{b}$$

where $F_{b}$ is the force vector of the vehicle to the road; $M_{b}$, $C_{b}$, and $K_{b}$ represent the mass matrix, damping matrix, and stiffness matrix of the road, respectively; ${\ddot{X}}_{b}$, ${\dot{X}}_{b}$, and $X_{b}$ represent the acceleration response, velocity response, and displacement response of the road, respectively.

The vibration equation of the vehicle–road coupling system can be obtained simultaneously by using Equations (9) and (10).

### 3.2. Newmark-β Iterative Algorithm

In order to solve the vehicle–road coupling system for numerical analysis, and considering the time-varying characteristics of the system, the iterative method is used to analyze the dynamic response process. The step-by-step iterative method of Newmark-β is adopted [35].

The dynamic response process of vehicle–road coupling system at t+Δt time satisfies Equation (11).

(11) $$M\cdot {\ddot{x}}_{t+\Delta t}+C\cdot {\dot{x}}_{t+\Delta t}+K\cdot x_{t+\Delta t}=F_{t+\Delta t}$$

In order to correct the assumed linear acceleration in the system, it is necessary to introduce α and β as control parameters to satisfy Equations (12) and (13).

(12) $$\ddot{x}=(1-\beta ){\ddot{x}}_{t}+\beta {\ddot{x}}_{t+\Delta t}$$

(13) $$\ddot{x}=(1-2\alpha ){\ddot{x}}_{t}+2\alpha {\ddot{x}}_{t+\Delta t}$$

where the control parameters α and β are satisfied with 0≤α≤0.5 and 0≤β≤1.

By assuming the initial state of the vehicle–road coupling system response, the time sequence information of the system response can be obtained iteratively.

**Figure 3:**
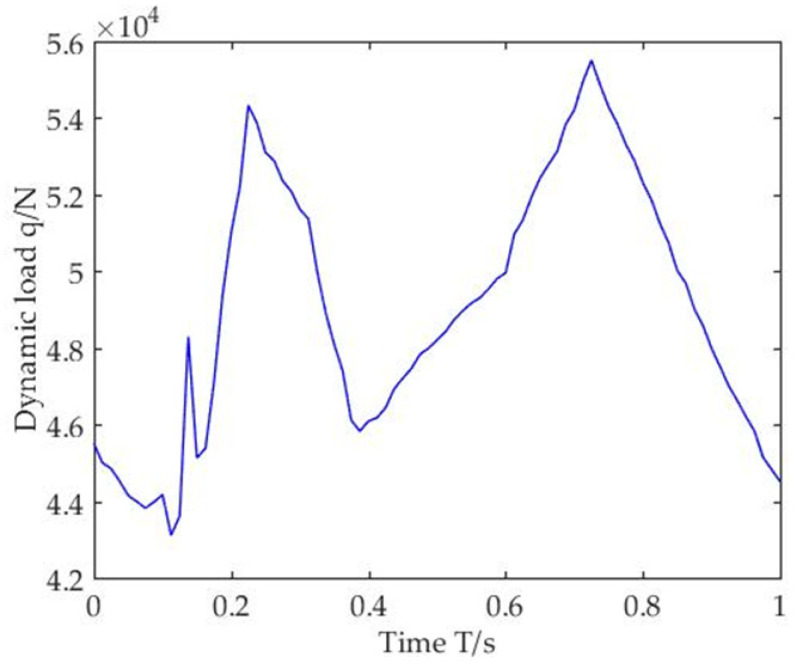
Vehicle tire dynamic load.

When the loaded vehicle is set to drive on the Class-B road at the speed of 20 m/s, and the values of α and β in the iterative algorithm are 0.25 and 0.5, respectively, then the dynamic tire load of the vehicle can be obtained, as shown in Figure 3.

## 4. Road Finite Element Model Analysis

### 4.1. Analysis of Road Structure and Parameters

According to the actual situation, this paper uses the ANSYS Workbench finite element software platform to establish the classical semi-rigid asphalt pavement model. In the process of research, certain assumptions need to be made about the model in order to combine the accuracy of the model and the simplicity of calculation. The asphalt pavement model established by the study is a multi-layer structure. The material of the same layer is homogeneous and continuous, and there is also a continuous system between each layers, so there is no void phenomenon [36].

**Figure 4:**
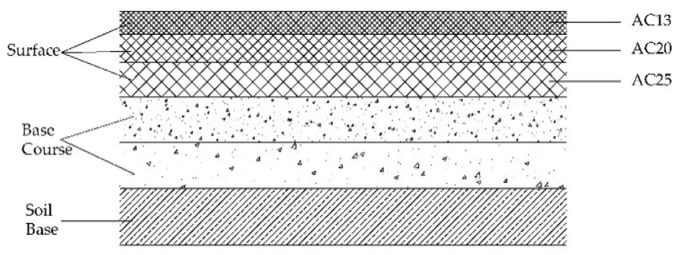
Schematic diagram of pavement structure.

On the basis of the above assumptions, combined with the theory of layered systems, a finite element model of a semi-rigid base asphalt pavement is established [37,38]. The specific composition of structural materials and the distribution of structural layers are shown in Figure 4.

### 4.2. Model Establishment and Analysis

**Figure 5:**
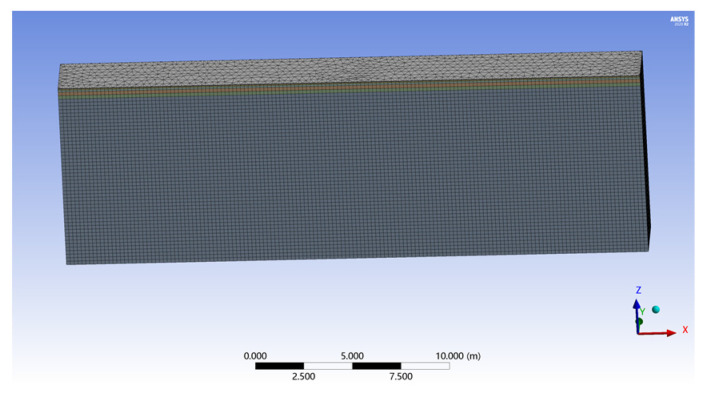
Road finite element model.

According to the above assumptions and road structure schematic diagram, the road finite element model established by the ANSYS Workbench finite element software platform is shown in Figure 5. The size of the model is 30 m (x direction), 3.5 m (y direction), and 10 m (z direction). The boundary condition is that the displacement is zero on the plane of x direction at 0 m and 30 m, zero on the plane of y direction at 0 and 3.5 m, and there is no z-direction displacement in the zonal 0 plane.

**Figure 6:**
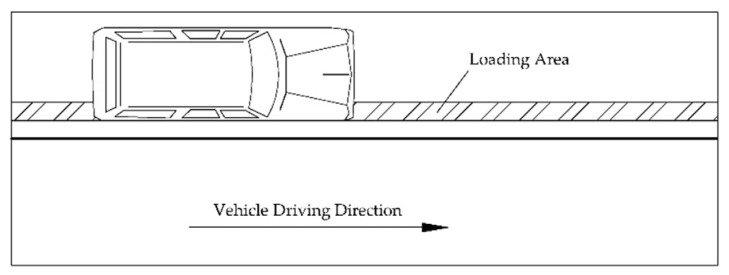
Schematic diagram of the vehicle’s driving trajectory.

When the vehicle is driving on the horizontal road, the road surface is mainly subjected to the vertical dynamic load from the vehicle [39]. This load is transmitted by the contact between the vehicle tire and the road surface. Suppose the vehicle is driving along the central axis of the road, and the contact area between the tire and the road surface is regarded as a rectangle [40]. In order to eliminate the boundary effect, the loading force is applied at x = 5 m of the finite element model, and stopped at x = 25 m. The schematic diagram of the vehicle track is shown in Figure 6.

### 4.3. Power Spectral Density Analysis

**Figure 7:**
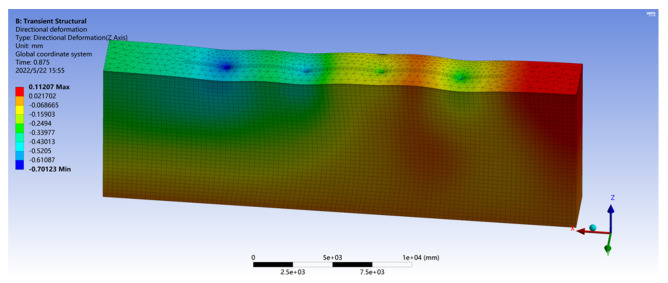
Road deformation map during vehicle driving.

Assuming that the vehicle passes through the loading area at a uniform speed of 20 m/s, combined with the vehicle tire dynamic load obtained by solving the vehicle–road dynamic model, the driving condition of the vehicle on the road is simulated by setting the load force in the road finite element model. The road surface deformation process when the vehicle is moving is shown in Figure 7.

**Figure 8:**
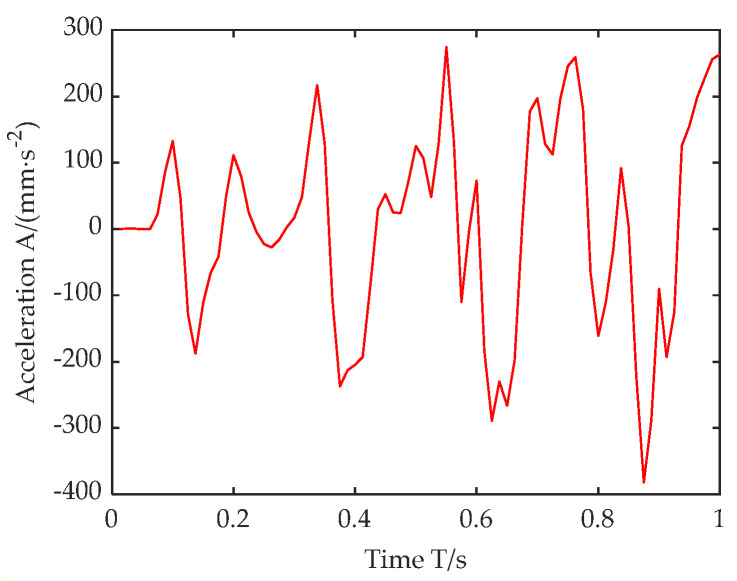
Roadside acceleration response curve.

In the road finite element model, by setting a virtual acceleration probe sensor on the roadside, the vibration acceleration response of the Z axis of the roadside observation point under the moving load of the vehicle is collected, as shown in Figure 8.

**Figure 9:**
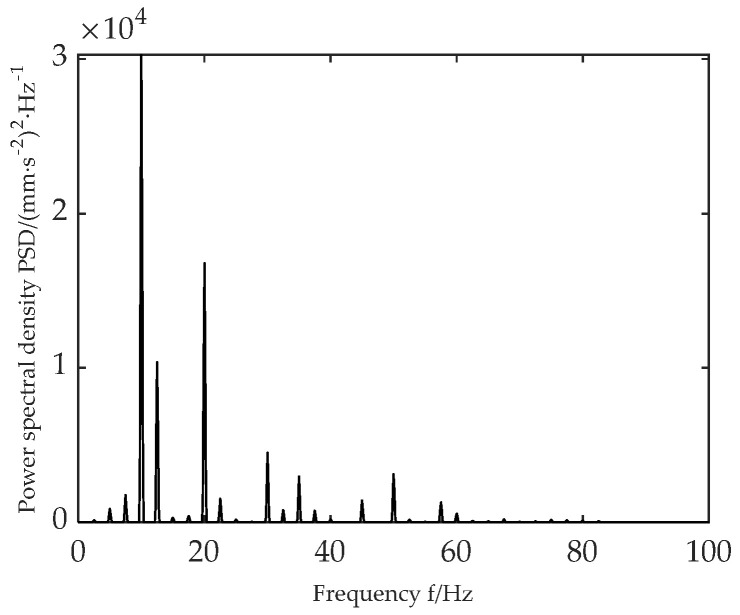
Roadside acceleration response spectral density.

A total of 20 groups of virtual acceleration probe sensors are set up in a certain area of the roadside. The acceleration data of the detection points are transformed using Fourier transform, and the power spectrum density of the roadside random vibration acceleration response is shown in Figure 9. As can be seen from Figure 9, the main frequency component of the vertical acceleration response on the roadside is between 5~30 Hz, and the peak value appears around 10 Hz. The experimental data are in good agreement with the measured data [41].

## 5. Fatigue Life Analysis of Equipment

### 5.1. Model Building

By taking the vehicle tire dynamic load obtained from the solution as the road surface load force, a virtual sensor is then setting up to collect the road surface vibration acceleration data. After the roadside acceleration spectral density is obtained by Fourier transform, the actual vibration excitation of the electromechanical equipment caused by the vehicle–road coupling is obtained. In order to analyze whether this vibration excitation will affect the reliability of the equipment, it is necessary to analyze the fatigue life of the highway electromechanical equipment under the influence of vibration excitation. However, due to the complex design structure of highway electromechanical equipment, its large volume, and its many components, it is generally difficult to analyze the overall dynamic response of such equipment. For this reason, this paper focuses on the study of the PCB, which has been widely used in electromechanical equipment, and analyzes its dynamic response and fatigue life.

The establishment of a finite element model, which can reflect the structural characteristics of the PCB, is the basis of using the finite element method to analyze its dynamic response. In finite element analysis, the common modeling methods of the PCB include: the simple modeling method, the total mass equivalent method, the stiffness equivalent method, the local equivalent method, and the direct finite element forming method [42]. This paper adopts the modeling method, which combines the total mass equivalent method and the direct finite element modeling method [43]. First, the 3D model is drawn according to the actual PCB using SolidWorks software. Then, appropriate simplification is carried out on the premise of retaining the basic features of the PCB. Through the analysis of the material properties of the PCB board, the substrate material is FR-4, and the parameters of other main materials, such as copper and electronic chips, are shown in Table 2 [44].

**Figure 10:**
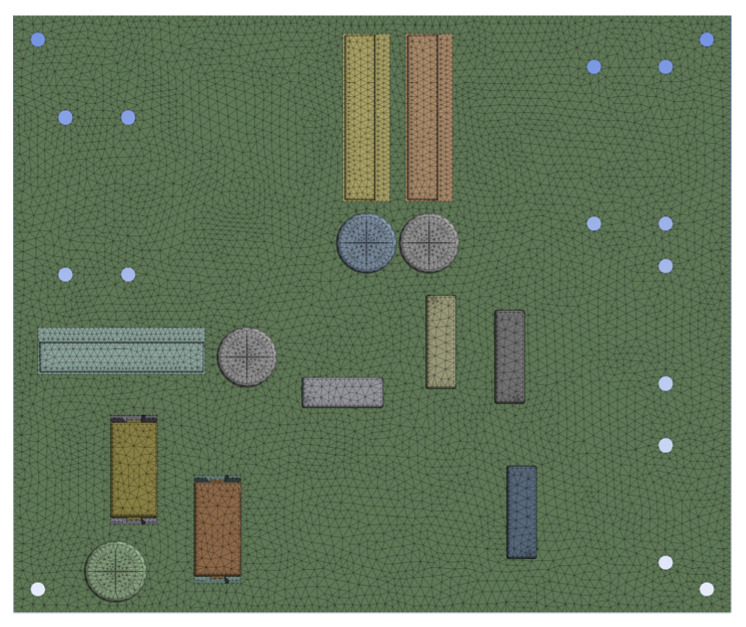
PCB simplified model.

Considering the PCB used in general highway roadside electromechanical equipment, which usually include a communication module, control module, voltage regulation module, filter module, etc., on the premise of retaining the basic characteristics of the original PCB—and comprehensively considering the requirements of computational efficiency—the tiny components, such as resistors, diodes, and small chips, are removed from the PCB, calculating the total weight of all the components, and distributing them on the PCB substrate. Due to the fact that some large components, such as the communication serial port of the communication module, are often fixed on the PCB substrate with bolts, these are also removed. The simplified PCB finite element model retains its own large weight, and at the same time, some control chips, large capacitors, voltage-regulating modules, etc., are connected to the PCB substrate via pin welding. The simplified PCB finite element mesh size of 1 mm is set, and the fixed constraints are imposed on the four corners. The simplified PCB model is shown in Figure 10.

Through statistics, the total mass of the established PCB model is determined to be 342 g, which is nearly consistent with the actual PCB weight. It can be concluded that there is no problem with the material definition in the process of modeling.

### 5.2. Fatigue Analysis

Through the random vibration analysis of the PCB finite element model, the vibration characteristics of PCB can be studied. Based on this, its dynamic response can be analyzed, and its reliability can be considered [45]. In the studies of random vibration, the environmental excitation under random vibration is generally given in the form of the power density spectrum, while the load distribution and dynamic response regarding the PCB are described by probability statistics. In the ANSYS Workbench platform, the response behavior of the PCB in the vehicle–road coupling vibration environment is simulated by inputting the power spectral density curve into the power spectral density (PSD) tool as the system excitation.

**Figure 11:**
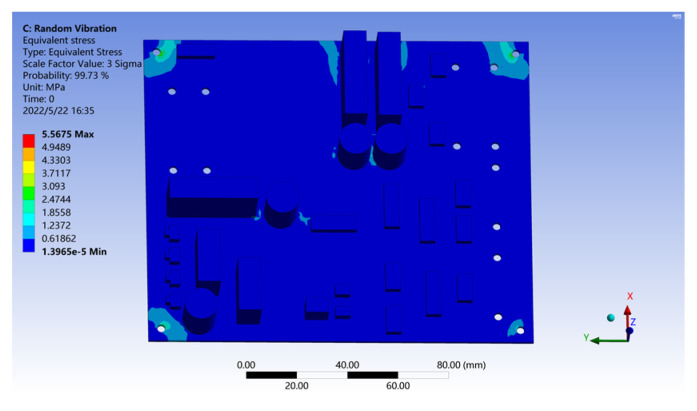
PCB equivalent stress results under roadside excitation.

By solving the model, the equivalent stress result of the PCB under vehicle–road coupling random vibration excitation is shown in Figure 11. The results show that within the probability range of 3 Sigma, the maximum equivalent stress caused by roadside random vibration excitation to the PCB, which is caused by vehicle–road coupling, is about 5.5675 MPa, which is much less than the tensile strength (340 MPa) of FR-4 material, and this stress is mainly distributed in the four-corner fixed hole. Therefore, it can be concluded that the random vibration excitation on the roadside does affect the structural strength of the PCB itself.

Most of the components in the PCB of the electromechanical equipment studied in this paper are packaged using a plastic ball grid array (PBGA). There is the research showing that the mechanical stress resistance of the PBGA solder joint is poor, and it cracks easily under continuous vibration stress, which leads to product failure [46]. At the same time, according to the life prediction method of electronic components proposed by Steinberg, the weakest link and the most likely failure in the PCB under dynamic loading will be the connection between the component and the substrate. If there is a failure here, the function of the PCB will be completely lost. If a fixed constraint is placed on the periphery of the PCB, a limit can be defined for the displacement somewhere in the center of the PCB panel, and the fatigue life of the PCB can be estimated for 20 million cycles in a random vibration environment. The calculation formula can be expressed as Equation (14).

(14) $$D=\frac{0.00022B}{Ehr\sqrt{L}}$$

where D is the maximum allowable displacement of the PCB (in); B is the length parallel to the edge of the PCB device (in); L is the length of the component on the PCB (in); h is the thickness of the PCB (in); E is the influence factor of the evaluated component on the PCB; and r is the position influence factor [47,48].

**Figure 12:**
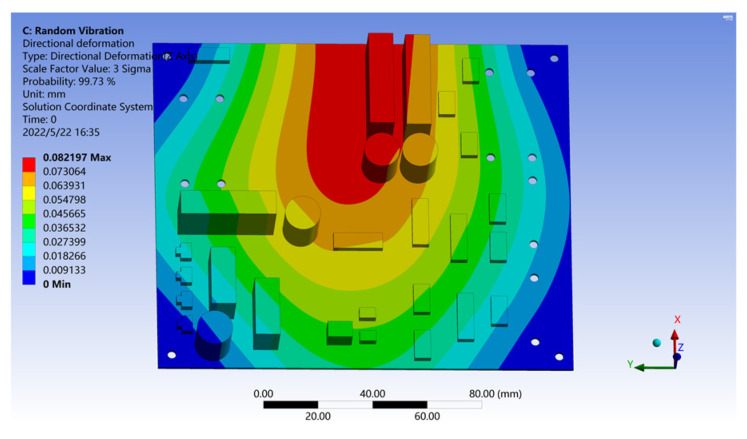
Deformation results of the PCB under random roadside vibration excitation.

From the solution results from the model, it can be seen that the maximum stress element on the PCB is located in the center of its upper side. After measurement, B = 5 in, h = 0.079 in, L = 1.38 in; make E = 2.25 and r = 0.707. Substitute Equation (14) to obtain D = 0.00527 in and =0.134 mm. The deformation analysis of the PCB is carried out by using the roadside acceleration response density spectrum as the vibration excitation, and the results are shown in Figure 12.

It can be seen from Figure 12 that under the random roadside vibration excitation, the Z deformation of PCB is only 0.0022 mm, which is much less than the D value, which means that the PCB can achieve a fatigue life of at least 20 million cycles under random roadside vibration excitation.

**Figure 13:**
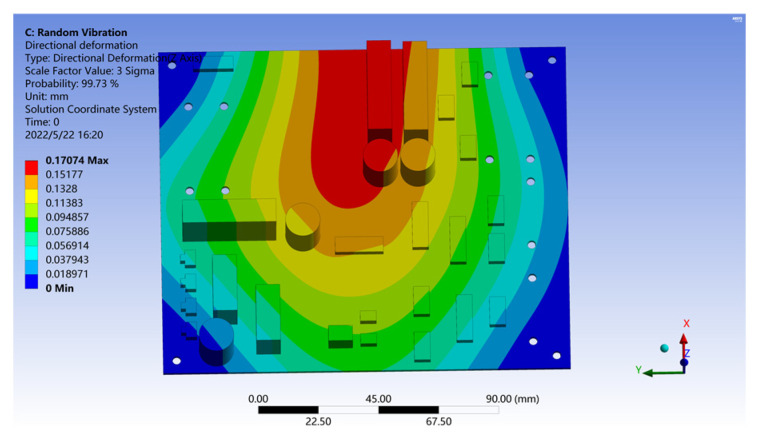
Deformation results of the PCB under random vibration excitation in the loading zone.

Because the roadside detection point is far away from the loading area of the vehicle—about 1.6 m away—the random vibration caused by vehicle–road coupling is actually greatly attenuated when it is transmitted to the roadside. We simulate the different driving habits of the vehicle by moving the position of the detection point forward; in which case, the vehicle is assumed to be driving close to the roadside. The power spectral density is obtained by the Fourier transform of the collected acceleration response data, and loaded into the PCB using the PSD tool. The results are shown in Figure 13, and they show that if the unattenuated vehicle–road coupled random vibration is used as the excitation source, the maximum Z-direction deformation of the PCB can reach 0.17 mm within the probability range of 3 Sigma, which is larger than the calculated displacement limit D. That is, in this environment, PCB cannot meet the fatigue life of at least 20 million cycles.

In summary, the research results show that if the vehicle is driving on the central axis of the lane, which means the coupling area between the vehicle and the road is relatively far away from the roadside electromechanical equipment, then the random vibration caused by the vehicle–road coupling will be attenuated enough that it cannot affect the structural characteristics of the PCB. However, if the vehicle on the highway is close to the roadside during driving, when the random vibration response caused by vehicle–road coupling acts directly on the PCB components of electromechanical equipment, without attenuation, its energy can cause greater deformation of the PCB, which may induce the components and substrates to break away from the PCB.

### 5.3. Optimal Design

**Figure 14:**
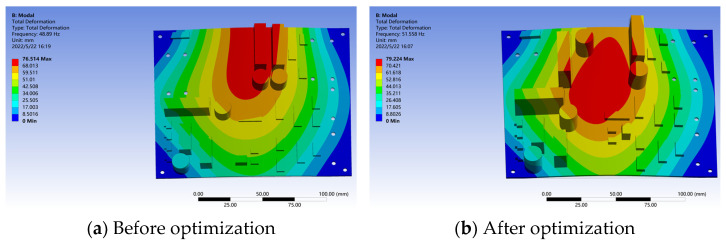
PCB first-order mode shape diagrams before and after optimization.

Through the PCB deformation result diagram studied in this paper, it can be seen that a larger element is located in the maximum deformation region of the PCB. At the same time, the research shows that the random vibration excitation near the vehicle–road coupling vibration source can cause damage to the PCB plate of the electromechanical equipment. Based on the above research results, this paper optimizes the overall design structure of the PCB by adjusting the position of its large components [49]. The comparison of the first-order mode shapes before and after optimization is shown in Figure 14.

As can be seen from Figure 14, the first-order modal frequency of the optimized PCB is increased by 5.4%, from 48.89 Hz to 51.56 Hz. At the same time, from the power density spectrum of Figure 9, it can be seen that the first-order mode of the optimized PCB avoids the main frequency region (10 Hz~50 Hz) of roadside vibration, thus greatly reducing the risk of resonance of the PCB board installed in the roadside electromechanical equipment.

**Figure 15:**
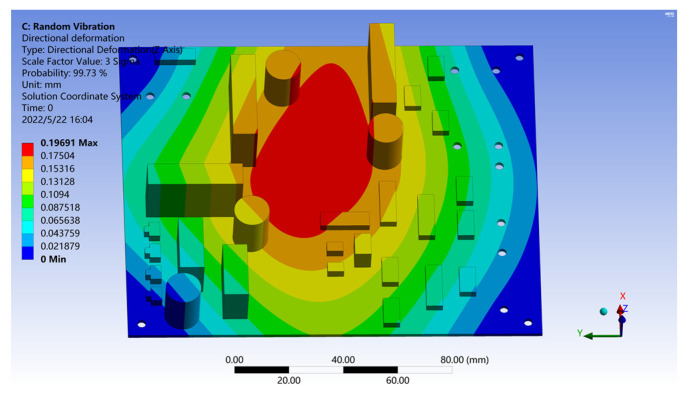
PCB deformation results after optimization.

The acceleration response density spectrum near the vehicle load loading area is introduced as the excitation, and the optimized PCB deformation result is obtained, as shown in Figure 15. The result shows that although the maximum deformation increases slightly after optimization, all the large components on the optimized PCB plate are distributed outside the deformation zone, thus effectively avoiding the risk of the large components breaking away from the joint with the PCB due to the large deformation of the PCB substrate, which could eventually lead to the loss of PCB functionality.

## 6. Conclusions

The purpose of this paper was to study whether roadside electromechanical equipment, after working in a complex traffic environment for a long period time, would be affected by vehicle–road coupling vibration, and the experimental results achieve this purpose. Through the finite element platform, this paper verifies the feasibility of studying the PCB reliability of highway electromechanical equipment by using roadside sensors to collect the pavement vibration data. The conclusions are as follows:

(1) When the vehicle trajectory is far from the roadside, due to the attenuation of random vibration caused by vehicle–road coupling, the structural characteristics of PCB are not affected, but if the vehicle is driving close to the roadside, because the roadside equipment is close to the loading area of vehicle–road interaction at this time, the random vibration caused by vehicle–road coupling will lead to greater deformation of the PCB. The experimental results show that the deformation value reaches 0.17 mm, which is higher than the maximum displacement of 0.134 mm allowed, as calculated by the Steinberg method. Large PCB deformation caused by vehicle–road coupling vibration can cause the connection between the component and the PCB substrate to be broken, resulting in the loss of the PCB function; thus, the electromechanical equipment will not operate properly.

(2) The PCB is optimized according to the fatigue analysis results. After optimization, the first-order modal frequency of the PCB is increased by 5.4%, and all the large components on the experimental PCB are distributed outside the large deformation area, which reduces the risk of breakage at the connection between the element and the PCB substrate.

The work done in this paper provides a theoretical basis for follow-up experiments. In the future, the collection of the actual vibration data from the highway pavement, by placing vibration sensors on the roadside, is planned. According to the experimental flow and method carried out in this paper, the data will then be used to analyze the influence of real random vibrations caused by vehicle–road coupling on the reliability of highway roadside electromechanical equipment.

## Figures and Tables

**Table 1:** Vehicle model parameters.

Material Name	Numerical Value
Partial mass of car body m1/Kg	4450
Partial mass of wheel m2/Kg	550
Suspension stiffness k1/(MN·m^−1^)	1
Wheel stiffness k2/(MN·m^−1^)	1.75
Suspension damping c1/(MN·s·m^−1^)	15
Wheel damping c2/(MN·s·m^−1^)	0

**Table 2:** Material properties.

Material Name	Density (kg/m^−3^)	Modulus of Elasticity (GPa)	Poisson’s Ratio
FR-4	1850	10	0.28
Cu	8940	120	0.35
Electronic Chip	2420	16	0.30

## Data Availability

Not applicable.

## References

[1] Sun W.-X., He T. (2022). Analysis on Intelligent Development of Expressway Mechanical and Electrical Operation and Maintenance Management. Highway.

[2] Qi H., Ganesan S., Pecht M. (2008). No-fault-found and intermittent failures in electronic products. Microelectron. Reliab..

[3] Fu Z.-J. (2018). Study on Vibration Law and Energy Spectrum Characterization of Asphalt Pavement under Vehicle-Road Coupling.

[4] Ekanthamoorthy J., Gnanasekaran V. (2020). Fatigue Estimation of Board Level ElectronicPackages under Random Vibrations. J. Xi’an Univ. Archit. Technol..

[5] Ye Z., Yan G., Wei Y., Zhou B., Li N., Shen S., Wang L. (2021). Real-Time and Efficient Traffic Information Acquisition via Pavement Vibration IoT Monitoring System. Sensors.

[6] Singh T., Sehgal S., Prakash C., Dixit S. (2022). Real-Time Structural Health Monitoring and Damage Identification Using Frequency Response Functions along with Finite Element Model Updating Technique. Sensors.

[7] Lu Q.-C., Zhang L., Xu P.-C., Cui X., Li J. (2022). Modeling network vulnerability of urban rail transit under cascading failures: A Coupled Map Lattices approach. Reliab. Eng. Syst. Saf..

[8] Ma X., Quan W., Dong Z., Dong Y., Si C. (2022). Dynamic Response Analysis of Vehicle and Asphalt Pavement Coupled System with the Excitation of Road Surface Unevenness. Appl. Math. Model..

[9] Zhang J., Yang S., Li S., Lu Y., Ding H. (2021). Influence of Vehicle-road Coupled Vibration on Tire Adhesion Based on Nonlinear Foundation. Appl. Math. Mech..

[10] Snehasagar G., Krishnanunni C.G., Rao B.N. (2020). Dynamics of Vehicle–pavement System Based on a Viscoelastic Euler–Bernoulli Beam Model. Int. J. Pavement Eng..

[11] Elnashar G., Bhat R.B., Sedaghati R. (2019). Modeling and Dynamic Analysis of a Vehicle-flexible Pavement Coupled System Subjected to Road Surface Excitation. J. Mech. Sci. Technol..

[12] Chen H.-Y., Ding H., Chen L.-Q. (2021). Research on Galerkin Truncation Convergence Order in Vehicle-road Coupled Vibration. Chin. J. Mech. Eng..

[13] Zhang J., Yang S., Li S. (2022). Study on Crack Propagation Path of Pavement Under Vehicle-road Coupled Vibration. Appl. Math. Model..

[14] Zini G., Betti M., Bartoli G. (2022). Experimental Analysis of the Traffic-induced-vibration on an Ancient Lodge. Struct. Control Health Monit..

[15] Arabi F., Gracia A., Delétage J.-Y., Frémont H. (2020). Effect of Thermal and Vibrational Combined Ageing on QFN Terminal Pads Solder Reliability. Microelectron. Reliab..

[16] Wang G., Zhang J., Kong X. (2020). Study on Passenger Comfort Based on Human–Bus–Road Coupled Vibration. Appl. Sci..

[17] Qin F., Bie X.-R., Chen S., An T. (2021). Fatigue Life Model of Leaded Solder Joints of Plastic Encapsulated Ball Grid Array Under Random Vibration Load. J. Vib. Shock.

[18] Rajaguru P., Lu H., Bailey C., Bella M. (2020). Modelling and Analysis of Vibration on Power Electronic Module Structure and Application of Model Order Reduction. Microelectron. Reliab..

[19] Wang J., Qin X., Liu Z., Ding G., Cai G. (2021). Experimental Study on Fatigue Degradation of Piezoelectric Energy Harvesters under Equivalent Traffic Load Conditions. Int. J. Fatigue.

[20] Muhammad N., Fang Z., Shoaib M. (2020). Remaining Useful Life (RUL) Estimation of Electronic Solder Joints in Rugged Environment Under Random Vibration. Microelectron. Reliab..

[21] Prashanth M.D. (2018). Vibration Analysis of Printed Circuit Boards: Effect of Boundary Condition. AIP Conf. Proc..

[22] Targosz J., Wiederek J. (2017). Drgania w Transporcie Drogowym i Ich Oddziaływania. Autobusy Tech. Eksploat. Syst. Transp..

[23] Du Y.-C., Liu C.-L., Wu D.-F., Zhao C. (2022). New Generation Intelligent Highway System Architecture Design. J. China Highw..

[24] Zacharakis I., Giagopoulos D. (2022). Vibration-Based Damage Detection Using Finite Element Modeling and the Metaheuristic Particle Swarm Optimization Algorithm. Sensors.

[25] Liu D.-W., Dai Z.-H., Chen Y., Chen H.-M. (2017). Dynamic Stress Response of Flexible Pavement Under Multi-wheel Dynamic Loads of Vehicles. China J. Highw. Transp..

[26] Zhang B.-Q. (2013). Coupling Vibration Analysis and Ride Comfort Evaluation of Vehicle-Road System.

[27] Li Y., Honeysuckle (2013). Study on the Interaction of Vehicle-asphalt Pavement Coupling System. Vib. Shock.

[28] Zhang F., Feng D.-C., Ling X.-C., Li Q.-L. (2015). Vertical Coupling Dynamic Model of Heavy-duty Vehicle-pavement-subgrade. China J. Highw. Transp..

[29] Du F., Ge X.-C., Chen X., Ding J.-Q. (2015). Study on Pavement Power Spectral Density Conversion and Roughness Modeling Theory. Vib. Test Diagn..

[30] Li Z., Au F.T.K. (2015). Damage Detection of Bridges Using Response of Vehicle Considering Road Surface Roughness. Int. J. Struct. Stab. Dyn..

[31] Ma X.-Y., Quan W.-W., Dong Z.-J., Si C.-L., Li S.-H. (2021). Dynamic Response Analysis of Vehicle Asphalt Pavement Under Random Unevenness Excitation. J. Mech. Eng..

[32] Zhang J.-L., Chen R.-F., Yao K. (2019). Analysis of the Influence of Road Surface Unevenness on Vehicle Vibration. J. Highw. Transp. Res. Dev..

[33] Liu X., Wang H., Shan Y. (2015). Construction of Road Roughness in Left and Right Wheel Paths Based on PSD and Coherence Function. Mech. Syst. Signal Process..

[34] Deng L., Chu H.-H., Wang W., Xu J. (2021). Influence of Vehicle-induced Fatigue Damage on the Reliability of Ultimate Bearing Capacity of Steel-concrete Composite Girder Bridges. China J. Highw. Transp..

[35] Pourzeynali S., Zhu X., Ghari Z.A. (2021). Comprehensive Study of Moving Load Identification on Bridge Structures using the Explicit Form of Newmark-β Method: Numerical and Experimental Studies. Remote Sens..

[36] Wang Y., Chao M., Sui C. (2014). Natural Frequency Sensitivity Analysis of a Road Model Based on the Finite Element Method. J. Eng. Sci. Technol. Rev..

[37] Karadag H., Firat S., Isik N.S. (2022). Determination of Permanent Deformation of Flexible Pavements using Finite Element Model. Građevinar.

[38] Pirmohammad S., Majd S.Y. (2020). Finite Element Analysis of Road Structure Containing Top-down Crack Within Asphalt Concrete Layer. J. Cent. South Univ..

[39] Ye Y., Cheng L., Yao H.-Q. (2021). Dynamic Response Analysis of Asphalt Pavement Under Different Interlayer Contact States. Highway.

[40] Liang C., Ji L., Mousavi H. Evaluation of Tire Traction Performance on Dry Surface Based on Tire-road Contact Stress. Proceedings of the 30th SIAR International Congress of Automotive and Transport Engineering.

[41] Lou M.-L., Tan G.-B. (2013). Measured Vibration Caused by Highway Traffic Operation and Its Attenuation Analysis. Chin. Q. Mech..

[42] Ren G., Li B., Li D. Modal Analysis of the Printed Circuit Board Based on Finite Element Method. Proceedings of the 2014 International Conference on Computer Science and Electronic Technology (ICCSET 2014).

[43] Feng Y.-X. (2018). Reliability Analysis and Experimental Research of a Product Circuit Board.

[44] Kalyani U.H., Wylie M. (2020). Modal Finite Element Analysis of PCBs and the Role of Material Anisotropy. Vibroeng. Procedia.

[45] Jayaraman S., Trikha M., Kamesh D. (2016). Response Spectrum Analysis of Printed Circuit Boards Subjected to Shock Loads. Procedia Eng..

[46] Zhao Z., Hu C., Yin F. Failure Analysis for Vibration Stress on Ball Grid Array Solder Joints. Proceedings of the IEEE 2018 19th International Conference on Electronic Packaging Technology (ICEPT).

[47] Kavitha M., Mahmoud Z.H., Kishore K.H., Petrov A.M., Lekomtsev A., Iliushin P., Salmani M. (2021). Application of Steinberg Model for Vibration Lifetime Evaluation of SN-AG-CU-based Solder Joints in Power Semiconductors. IEEE Trans. Compon. Packag. Manuf. Technol..

[48] An T., Qin F., Zhou B., Chen P., Dai Y., Li H., Tang T. (2019). Vibration Lifetime Estimation of PBGA Solder Joints Using Steinberg Model. Microelectron. Reliab..

[49] Liu F., Lu Y., Wang Z. (2015). Numerical Simulation and Fatigue Life Estimation of BGA Packages Under Random Vibration Loading. Microelectron. Reliab..

